# Prevalence of Monoclonal B Lymphocytosis in First-Degree Relatives of Chronic Lymphocytic Leukemia Patients in Turkey

**DOI:** 10.4274/tjh.2013.0288

**Published:** 2015-02-15

**Authors:** Taner Demirci, Zeynep Arzu Yeğin, Nevruz Kurşunoğlu, Zeynep Yılmaz, Elif Suyanı, Zübeyde Nur Özkurt, Münci Yağcı

**Affiliations:** 1 Gazi University Faculty of Medicine, Department of Internal Medicine, Ankara, Turkey; 2 Gazi University Faculty of Medicine, Department of Hematology, Ankara, Turkey

**Keywords:** Monoclonal B lymphocytosis, prevalence, Chronic lymphocytic leukemia, First-degree relatives

## Abstract

**Objective:**

Monoclonal B lymphocytosis (MBL) is considered to be a precursor state for chronic lymphocytic leukemia (CLL). This study was planned to evaluate the MBL prevalence in first-degree relatives of CLL patients in Turkey, which is considered to be an ethnic and geographic bridge between the Eastern and Western worlds.

**Materials and Methods:**

A total of 136 volunteers [median age: 40 (17-77) years; male/female: 60/76] from 61 families were included. Flow cytometry analysis by 4-colour staining was used for MBL diagnosis.

**Results:**

MBL was demonstrated in 17 cases (12.5%). A total of 14 cases (10.3%) were classified as CLL-like MBL, while 3 (2.2%) exhibited a non-CLL-like phenotype. The prevalence of MBL was 12.72% in subjects aged less than 40 years, 12.28% in subjects between 40 and 60 years, and 40% in subjects over 60 years, without statistical significance (p>0.05). A total of 115 cases were evaluated for intermarriage, which was observed in 19 cases (16.5%). The prevalence of MBL did not differ based on intermarriage status (p>0.05).

**Conclusion:**

The current report is the first MBL prevalence study in a Eurasian population that demonstrates a similar distribution pattern of MBL in Anatolian CLL kindreds. Further efforts should be made to refine our understanding of the natural history and clinical outcomes of MBL.

## Abstract (tr)

**Amaç:**

Monoklonal B lenfositozun (MBL) kronik lenfositik lösemi (KLL) için öncül bir durum olduğu düşünülmektedir. Bu çalışma doğu ve batı dünyası arasında etnik ve coğrafi köprü olduğu düşünülen Türkiye’de KLL hastalarının birinci derece akrabalarında MBL sıklığının araştırılması için tasarlanmıştır.

**Gereç ve Yöntemler:**

Çalışmaya 61 aileden olmak üzere toplam 136 gönüllü [ortalama yaş 40 (17-77); erkek/kadın: 60/76] dahil edilmiştir. MBL tanısı içi dört farklı boyama ile akım sitometri analiz yöntemi kullanılmıştır.

**Bulgular:**

Monoklonal B lenfositoz 17 (%12,5) olguda tespit edilmiştir. Olguların 14’ü (%10,3) KLL benzeri MBL fenotipi olarak sınıflandırılırken kalan 3 olgu (%2,2) KLL dışı MBL olarak sınıflandırılmıştır. MBL prevalansı 40 yaş altı olgularda %12,72, 40-60 yaş arası olgularda %12,28, 60 yaş üstü olgularda %40 olarak gözlenmiştir ve istatistiksel olarak anlamlı değildir (p>0,05). Akraba evliliği yönünden 115 olgu değerlendirilmiş olup bunlardan 19’unda (%16,5) akraba evliliği öyküsü mevcuttu. MBL prevalansı akraba evliliği durumu ile farklılık göstermiyordu (p>0,05).

**Sonuç:**

Bu araştırma Avrasya topluluğunda yapılan ve aynı etnik özellikleri taşıdığı Anadolu kökenli KLL hastalarının akrabalarında MBL dağılım şeklini gösteren ilk MBL prevalans çalışmasıdır. MBL’nin doğal gelişimini ve klinik sonuçlarını daha iyi anlayabilmemiz için daha fazla çaba harcanmalıdır.

## INTRODUCTION

Chronic lymphocytic leukemia (CLL) is the most common haematological malignancy, which accounts for 30% of all leukemias in the Western world [1,2,3,4,5,6,7,8]. Despite knowledge of the classical risk factors of male sex, advanced age, white race, and a family history of CLL, the aetiology and pathogenesis remain largely unknown [3,4,5,6,7,9]. In previous reports, the presence of familial clustering in CLL has been widely confirmed. Approximately 12% of patients with CLL report a family history of a lymphoproliferative disease, while 6-9% have a relative who has also CLL [2,3,4,10]. Based on epidemiological studies, first-degree relatives of CLL patients have 3-8 times greater risk for the development of CLL [2].

Diagnostic criteria for CLL are the presence of monoclonal B lymphocytes with CD5, CD19, and CD23 expression, and weak or no expression of CD20, CD79b, FMC7, and surface immunoglobulin. The monoclonality should be represented in the majority of leukocytes with an absolute lymphocyte count (ALC) of >5x109/L [7,11,12,13,14].

Monoclonal B lymphocytosis (MBL) is considered to be a precursor state for CLL, similarly to the association of monoclonal gammopathy of undetermined significance (MGUS) and plasma cell myeloma. According to the current data, CLL is accepted to be preceded by a MBL state. A prediagnostic B cell clone was demonstrated in 98% of CLL patients 77 months before the CLL diagnosis [7,9,15]. MBL is defined as the presence of a clonal B cell population in the context of an absolute B cell count (ABCC) of <5x109/L, no history of autoimmune disease, and no evidence of lymphadenopathy and organomegaly on physical examination [3,7,9,12,13,14,15,16,17,18,19]. Monoclonal B lymphocytosis is subclassified into 3 immunophenotypic categories: CLL-like (CD5+23+), atypical CLL (CD5+20bright), and non-CLL (CD5-). The most common MBL subtype is CLL-like MBL, while atypical and non-CLL types of MBL account for only 15%-30% of all MBL cases [7,9,13,14,15,16]. MBL and CLL share a similar genetic profile. Deletion of 13q and trisomy 12 are identified in MBL at similar frequencies to CLL, but the higher risk abnormalities such as deletions of 11q and 17p appear to be rare in MBL. To our knowledge, 88%-96% of MBLs have mutated immunoglobulin heavy variable group genes, with intraclonal heterogeneity similar to that of CLL [2,13,15].

The prevalence of MBL is dependent largely on the characteristics of the study population and the sensitivity of flow cytometry methods. The lack of standardisation in this context complicates determination of the true prevalence, as highly sensitive flow cytometry methods reveal higher estimates. As a result, prevalence in the general adult population is reported to be 0.1%-14%, indicating a wide range [14,17]. MBL is more common in CLL families. The prevalence of MBL among first-degree relatives of CLL patients ranges between 12% and 18% [1,10,14].

At present, detailed information on epidemiological characteristics of MBL is not available. In particular, MBL status in Asia or Africa is entirely unknown, although CLL is reported to be less common in Eastern parts of the world [2,3,4,7]. This study was projected to evaluate the MBL prevalence in first-degree relatives of CLL patients in Turkey, which is considered to be an ethnic and geographic bridge between the Eastern and Western worlds.

## MATERIALS AND METHODS

A total of 136 first-degree volunteer relatives [median age: 40 (17-77) years; male/female: 60/76] from 61 CLL families were included. We utilised the recently defined diagnostic criteria for MBL [12]: 1) detection of a disease-specific immunophenotype or an overall kappa (κ)/lambda (λ) ratio of >3:1 or <0.3:1, 2) stable monoclonal B cell population over a 3-month period, and 3) absence of lymphadenopathy, organomegaly, and autoimmune or infectious diseases, and B lymphocyte counts of <5x109/L. MBL was classified as CLL-like (CD5+23+) or non-CLL (CD5-) types.

Flow cytometry analysis by 4-colour staining was performed using peripheral blood collected into EDTA. All samples were analysed on a FACSCalibur flow cytometer (Becton Dickinson). Monoclonal antibodies IgM fluorescein isothiocyanate (FITC), IgD phycoerythrin (PE), and IgG FITC were purchased from BD Pharmingen and all the rest from Becton Dickinson. A total of 200,000 events per tube were acquired. Sequential gating strategy was used as previously described [3,20].

**Figure 1:**
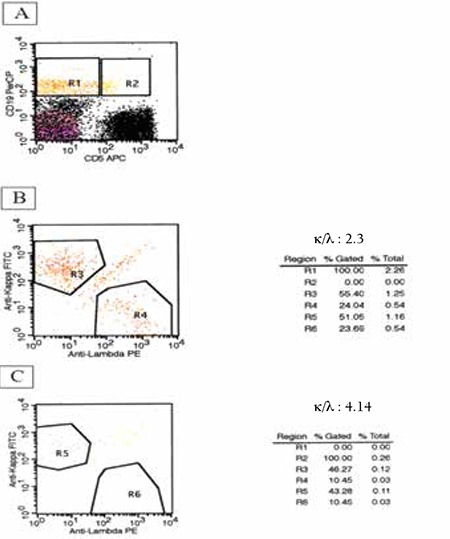
A) Evaluation of CD5 and CD19 expressions by biparametric graphics. R1 and R2 represent CD5-CD19+ and CD5+CD19+ cells, respectively. B) R3 shows CD5-CD19+κ+ and R4 shows CD5-CD19+λ+ cells. C) R5 shows CD5+CD19+κ+ and R6 shows CD5+CD19+λ+ cells.

A 2-step analysis method was used for MBL diagnosis. The initial panel consisted of CD5 allophycocyanin (APC) and CD19 peridin-chlorophyll protein (PerCP)/anti-κ FITC/anti-λ PE. Cells were first evaluated by biparametric graphics based on CD5 and CD19 expressions (Figure 1A). R1 and R2 represented CD5-CD19+ and CD5+CD19+ cells, respectively. Selected cells on R1 and R2 were then analysed for κ and λ expressions. R3/R5 and R4/R6 showed κ- and λ-positive cells, respectively (Figures 1B and 1C). Monoclonality was detected by light chain restriction, which was defined as κ/λ of >3:1 or <0.3:1. Whole blood count and second panels were performed in cases of B cell clonality in the initial panel.

The second panel was arranged based on the presence of lymphocytosis. If lymphocytosis was detected, the panel included CD3 FITC/CD3 control PE/CD19 PerCP; CD20 FITC/CD5 PE/CD19 PerCP; CD10 FITC/CD38 PE/CD19 PerCP; FMC7 FITC/CD22 PE/CD19 PerCP; CD11a FITC/CD23 PE/CD19 PerCP; IgM FITC/IgD PE/CD19 PerCP; and IgG FITC/CD79b PE/CD19 PerCP. If lymphocyte count was found to be normal, the analysis was switched to CD20 FITC/CD79b PE/CD19 PerCP; FMC7 FITC/CD23 PE/CD19 PerCP; and CD5 APC profile.

The study was approved by the Ethics Committee of the Turkish Ministry of Health and informed consent was received from all participants.

## Statistical Analysis

Statistical analysis of the data was performed with SPSS 15 (SPSS Inc., Chicago, IL, USA). Continuous variables were presented as median values, whereas categorical variables were presented as frequencies and percentages. Differences between categorical variables were evaluated with chi-square or Fisher’s exact test. Continuous variables were compared by Mann-Whitney U test for 2 independent groups or Kruskal-Wallis test for 3 or more groups. P-values of less than 0.05 were considered to be statistically significant.

## RESULTS

**Figure 2:**
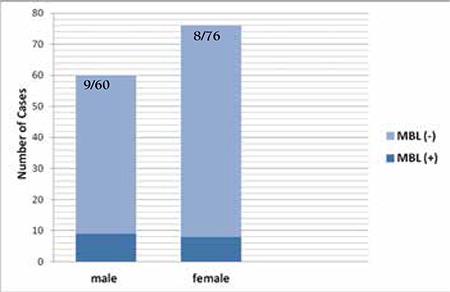
Monoclonal B lymphocytosis in male and female subjects.

MBL was demonstrated in 17 cases (12.5%). A total of 14 cases (10.3%) were classified as CLL-like MBL, while 3 (2.2%) exhibited the non-CLL-like phenotype. Median ALC was found to be 2.2x109/L (1.5-3.9) in MBL cases and 2.25x109/L (1.5-3) in normal subjects (p>0.05). Median ABCC in MBL cases was 1.48x109/L (0.3-2.8). Prevalence of MBL was not statistically different in male and female subjects (15% vs. 10.5%, respectively) (p>0.05) (Figure 2).

A total of 115 cases were evaluated for intermarriage, which was observed in 19 cases (16.5%). The prevalence of MBL did not differ based on intermarriage status (p>0.05). 

The geographic distribution of the 115 subjects was also evaluated. A total of 51 (44.3%) were from Central Anatolia, 2 (1.7%) from West Anatolia, 4 (3.5%) from South Anatolia, 19 (16.5%) from East Anatolia, 3 (2.6%) from South-East Anatolia, 34 (29.6%) from North Anatolia, 1 (0.9%) from North-West Anatolia, and 1 (0.9%) from Cyprus. MBL was more common in South (25%) and Central (17.6%) Anatolia. No MBL cases from North, West, and South-East Anatolia or Cyprus were found. The difference in MBL prevalence among geographic regions of Turkey was not found to be significant (p>0.05). Characteristics of the studied subjects are detailed in Table 1.

**Figure 3:**
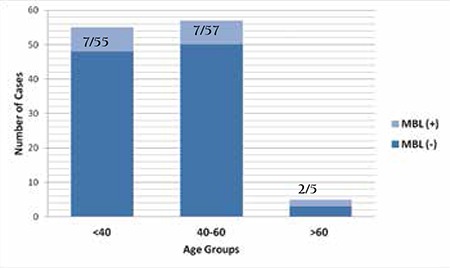
Monoclonal B lymphocytosis among age groups.

As the prevalence of MBL is indicated to vary based on age distribution [14,17], a total of 117 subjects including 16 MBL cases were divided into age groups defined as <40 years, 40-60 years, and >60 years. The prevalence of MBL was 12.72% (7/55) in subjects aged less than 40 years, 12.28% (7/57) in subjects between 40 and 60 years, and 40% (2/5) in subjects over 60 years, without statistical significance (p>0.05) (Figure 3).

## DISCUSSION

The present study investigated the prevalence of MBL in 136 first-degree relatives from 61 families of patients with CLL. MBL was demonstrated in 17 cases (12.5%). A total of 14 cases (10.3%) were classified as CLL-like MBL, while 3 (2,2%) displayed the non-CLL phenotype.

Variations in MBL prevalence can be attributed to study design, study population, and sensitivity of flow cytometric techniques [16]. Despite the variability in prevalence estimates, it is obvious that MBL is more common among the elderly and first-degree relatives of CLL patients. The prevalence of MBL in our cohort seems to confirm the previously reported data [1,2,3,7,10,21]. As intermarriage is frequently encountered in certain regions of Anatolia, we did analyse the possible impact of intermarriage on MBL prevalence in CLL families. However, we did not find any difference in MBL prevalence between families with and without intermarriage.

The reported prevalence of CLL-like MBL has increased significantly over time, representing up to 85% of all MBL cases. However, even in studies using very sensitive flow cytometry methods, the prevalence of CD5- MBL is considered to be around 2.3%, in concordance with our results [19,21]. The lack of large clinical series for CD5- MBL might be due to less peripheral blood involvement of lymphoproliferative diseases other than CLL.

The age and sex distribution of MBL is similar to that of CLL, as prevalence correlates with a male predominance and older age [15,21,22]. We also demonstrated an increase in MBL prevalence in elderly subjects, without statistical significance. This insignificance might be explained by the small sample size, as there were only 5 individuals who were >60 years in our study group. Similarly, we did not find any significant difference in MBL prevalence between males and females.

The clinical subgroups of CLL-like MBL can be defined as clinical MBL and low-count MBL (defined as <0.15x109 MBL cells/L). Clinical MBL refers to the MBL cases presented with lymphocytosis. The second category is represented by the MBLs discovered while screening [18,22]. Rawstron et al. investigated 1520 subjects with a normal blood count and 2228 subjects with lymphocytosis. A total of 185 subjects with CLL-like MBL and lymphocytosis were monitored for a median of 6.7 years. Progressive lymphocytosis occurred in 51 (28%), progressive CLL developed in 28 (15%), and chemotherapy was required in 13 (7%). The ABCC was the only independent prognostic factor associated with progressive lymphocytosis. Treatment requirements developed in subjects with clinical MBL at a rate of 1.1% per year, which is similar to the rate of progression to myeloma seen in patients with MGUS [13]. Median ALC was found to be 2.2x109/L (1.5-3.9) and median ABCC was 1.48x109/L (0.3-2.8) in our MBL cases. We could not make a comment on the outcome of MBLs in this study, as follow-up data are not currently available.

Rossi et al. compared 123 clinical MBL and 154 Rai 0 CLL patients according to clinical and biological profiles. The best B cell thresholds for the lowest and highest risk of progression to CLL were respectively defined as <1.2x109/L and >3.7x109/L [18]. Shanafelt et al. identified a B cell threshold of 11x109/L for the best prediction of survival in a study of 459 patients with a clonal CLL-like cell population. Similarly, Molica et al. investigated 1158 patients with newly diagnosed Binet A CLL and identified an ALC of 11.5x109/L and ABCC of 10x109/L as the best B cell thresholds for treatment requirement [15,23,24]. Generally, the use of ABCC rather than ALC for the diagnosis of CLL or MBL is recommended; however, a specific cut-off value that could be used for the discrimination of these entities has not been defined yet. Definite comment about the progression risk cannot be made, as MBL patients were not prospectively identified or followed [7,9,13].

The current report is the first MBL prevalence study in a Eurasian population that demonstrates a similar distribution pattern of MBL in Anatolian CLL kindreds. Further efforts should be made to refine our understanding of the natural history and clinical outcomes of MBL.

## Figures and Tables

**Table 1:**
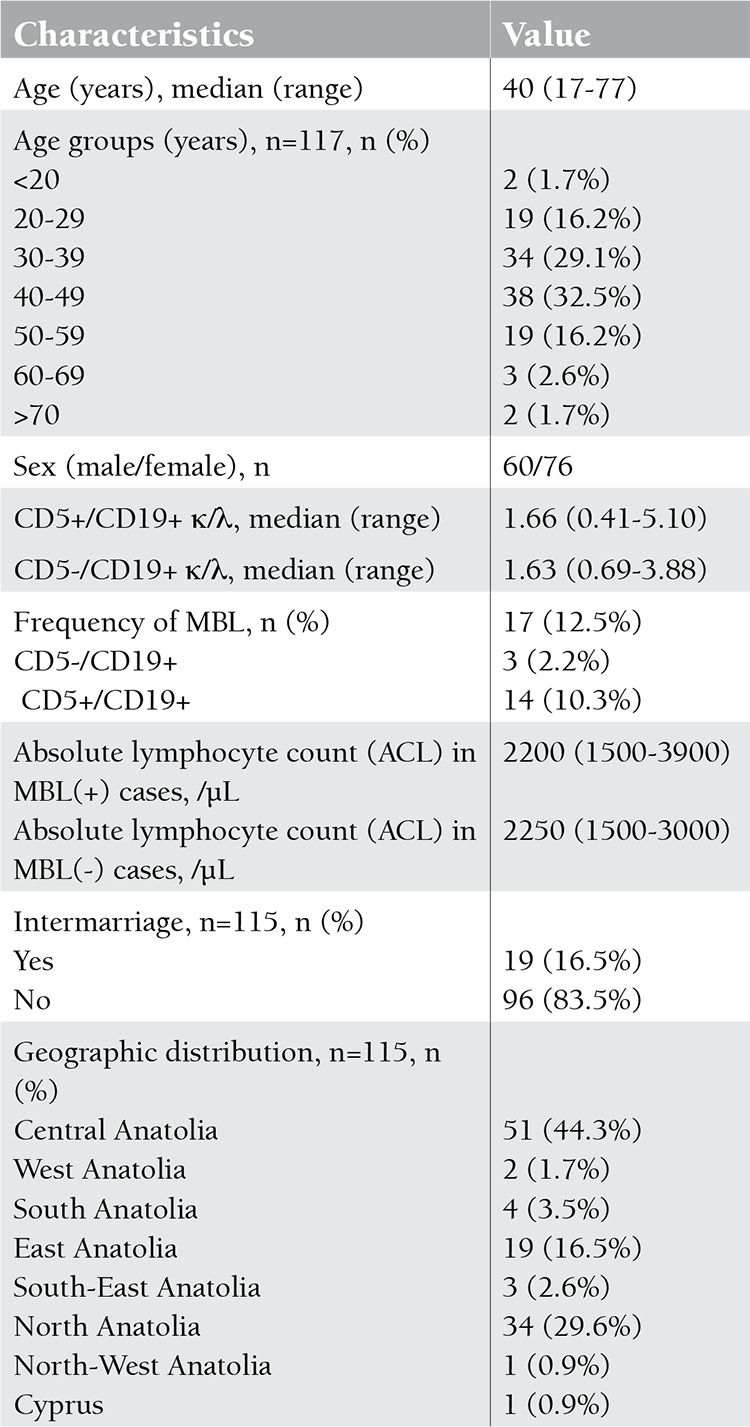
Characteristics of the studied subjects.
